# Monitoring the post-match neuromuscular fatigue of young Turkish football players

**DOI:** 10.1038/s41598-022-17831-7

**Published:** 2022-08-16

**Authors:** Zeki Akyildiz, Yücel Ocak, Filipe Manuel Clemente, Yasar Birgonul, Mehmet Günay, Hadi Nobari

**Affiliations:** 1 Faculty of Sport Sciences, Gazi University, Ankara, Turkey; 2 Faculty of Sport Sciences, Afyon Kocatepe University, Afyonkarahisar, Turkey; 3 Escola Superior Desporto e Lazer, Instituto Politécnico de Viana do Castelo, Rua Escola Industrial e Comercial de Nun’Álvares, 4900-347 Viana do Castelo, Portugal; 4 Delegação da Covilhã, Instituto de Telecomunicações, 1049-001 Lisboa, Portugal; 5 Faculty of Sport Sciences, University of Extremadura, 10003 Cáceres, Spain; 6 Department of Motor Performance, Faculty of Physical Education and Mountain Sports, Transilvania University of Braşov, 500068 Braşov, Romania; 7 Department of Exercise Physiology, Faculty of Educational Sciences and Psychology, University of Mohaghegh Ardabili, Ardabil, 56199-11367 Iran

**Keywords:** Physiology, Health occupations

## Abstract

Neuromuscular fatigue tests have been used in previous studies to organize post-match training programs and to minimize injuries. The aim of this study is to describe the neuromuscular fatigue that occurs after a football match and to examine the relationship between internal and external load values in the match and fatigue parameters obtained at different time intervals. Twenty male U19 academy league soccer players (age: 19; height: 181.3 ± 4.3; weight: 73.4 ± 6.7) participated in the study. The countermovement jump (CMJ) test was applied to the players 24 h before, as well as 24, 48, 72, 96, and 120 h after a football match. During the CMJ tests, the maximum velocity of each player during the jump was recorded by using the GymAware linear position transducer. The CMJ maximum velocity values 24 h before and 24 h after the match, as well as the CMJ height values (Cohen’s d: 1.210; p < 0.001), were statistically different from the values recorded 24 h before and 24 and 48 h after the match (Cohen’s d: 1.578; p < 0.001; Cohen’s d: 0,922; p < 0.009). The correlation values were not statistically significant. The results suggest, CMJ height and CMJ maximum velocity values, which determine neuromuscular fatigue after a football match, can be used by practitioners to display post-match neuromuscular fatigue measurements.

## Introduction

Football is considered a high-intensity interval sport with an unprecedented increase in high impulsive actions occurring during match play observed over the past decade^1^. Football players today experience an increase in the physical demands of matches due to short recovery times between matches and high neuromuscular demands^2^. Increasing demands in match and congested fixtures can cause temporary fatigue during matches^3,4^. Increased performance needs and recovery problems mean longer times are needed to fully recover^5,6^.

After eliminating the fatigue of a match played on the weekend, detailed plans need to be formed regarding the technical, tactical, and physical deficiencies of players via training until the next match is played the next weekend and; at the same time, players must be made ready for the next match with the most appropriate performance^7^. In some cases, there is not even a week's recovery period between games. Teams are exposed to fatigue due to congested fixtures and the team's playing in different leagues^3,4^. For this reason, observing neuromuscular fatigue in players is crucial for sports scientists and trainers to organize their weekly training programs correctly and to protect players from injuries^7,8^.

Neuromuscular fatigue has been defined as any exercise-induced reduction in maximal voluntary force or strength produced by a muscle or muscle group in humans^9,10^. Traditionally, neuromuscular fatigue has been studied using isolated forms of isometric, concentric, and eccentric movements^9^. However, recent evidence suggests that combining movements involving the stretch–shortening cycle (SSC) enables a more in-depth specific investigation of neuromuscular fatigue^11–13^. Movements involving SSC include metabolic, mechanical, and neural components of fatigue with impaired yawn reflex activation^13^. SSC involves a pre-activated muscle that is commonly used when one performs activities involving different stages of running or jumping, which is stretched first and then shortened^12^. Recovery after impaired SSC function takes place in two stages: (a) a significant reduction in SSC function immediately after exercise and (b) a phase of transient improvement followed by a subsequent decrease in performance, resulting in the highest decrease in SSC function 48–72 h after exercise^5,12–15^.

The countermovement jump (CMJ) test is widely used among soccer players and team players to measure neuromuscular fatigue^16–18^. CMJ decreases in direct proportion to decreases in SSC, thus reflecting neuromuscular fatigue^19–21^ also CMJ test is valid, reliable, and practical^22^ known to be a strong predictor of neuromuscular fatigue^23^. In addition, the validity and sensitivity of the CMJ test are high^24^, and it is potentially practical for detecting and measuring fatigue in field conditions^18,22^. Further, many studies have found that CMJ testing reflects neuromuscular fatigue^12,14^. Several studies have used CMJ tests to measure neuromuscular fatigue among football players^16,17,25–30^. In these studies, neuromuscular fatigue after matches was examined using the CMJ test^16,17,25–30^.

A better understanding of neuromuscular responses to soccer matches can improve individualized post-match strategies, reduce the risk of residual and cumulative fatigue, and reduce the rate of musculoskeletal injury. The current research systematically examined the time course of players’ neuromuscular responses at different time intervals before and after a football match. At the same time, this research examined the relationship between the internal and external training load values during the match and the changes in the fatigue variables according to time.

Therefore, the aims of this study are to examine the differences in the neuromuscular fatigue of football players 24, 48, 72, 96, and 120 h after a match and to investigate the relationship between internal and external training load values during a match and temporal changes in fatigue parameters. The findings of this study provide practical information to sports scientists and coaches about the training programs and recovery protocols of players. The findings of this study provide new information to researchers and practitioners by revealing differences in CMJ height and CMJ maximum velocity values, which are used to detect neuromuscular fatigue after a football match, and by investigating the relationship between internal and external training load variables during a match.

## Methods

### Experimental approach to the problem

An experimental study was carried out among the players of a football team. The study examined the neuromuscular fatigue that occurs in players during matches at different time intervals, as well as the relationship between internal and external training load variables in matches and neuromuscular fatigue at different times. The study was designed during a normal match period week in the 2020/2021 season. The tests were carried out during the regular match period of the season. All matches in the study were conducted under normal field conditions. The purposes of the study were to examine the differences between the pre-match baseline values and the post-match values at 24, 48, 72, 96, and 120 h, as well as to investigate the relationships between internal and external load demands and fatigue values during matches.

In the session 24 h before the match day, the height and weight of each athlete were measured. After the anthropometric measurements were taken, the players performed three CMJ repetitions. On the day of the match, the players were made to jump three times during the CMJ test. Immediately after the test, the players participated in a friendly match consisting of 90-min (2 × 45 min) periods and played under official field conditions.

**Figure 1:**
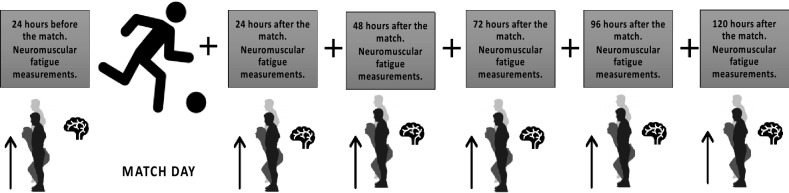
Plan structure of the experimental study protocol.

During the match, the players’ heart rate, accelerations, and distance traveled were measured with the help of Polar Pro Team GPS (Finland). The CMJ height, and CMJ maximum velocity values obtained from the CMJ tests at the 24th hour, 48th hour, 72th hour, 96th hour, and 120th hour after the match were recorded. The total length of this study was one week. The structure of the experimental study protocol is shown in Fig. 1.

### Participants

Twenty football players (age: 19; height: 181.3 ± 4.3; body mass: 73.4 ± 6.7) who played in the U19 Academy League voluntarily participated in the study. The criteria for inclusion in the study were at least 3 years of football history and no injury. Players who did not have a football background for 3 years, participated in heavy exercise before the tests and had any injuries were excluded from the tests in the study. 20 people participated in the study and all participants met the participation criteria. All participants consisted of football players who train regularly (5 days a week/1.5–2 h). All 20 participants performed the tests as part of the normal training program week and were familiar with the pre-study procedures. Within 24 h prior to the match, participants were encouraged to maintain adequate hydration and a balanced diet. This study was carried out in accordance with the Principles of the Declaration of Helsinki. After all the details of the study were explained to the participants, they were asked to fill in a written informed consent form. All the players participating in the study participated voluntarily. An application was made to the ethics committee of Afyon Kocatepe University for the study and the consent of the ethics committee, dated 19.01.2021, numbered 26.01.2021–3764, was obtained.

### Countermovement jump tests

The neuromuscular fatigue of the participants was determined using the countermovement jump (CMJ) test. Research reports that the CMJ test is a valid and reliable^18,20,22,31^. CMJ measurements were conducted prior to the training session. Players participated in a 10-min standardized warm-up prior to the test. This warm-up consisted of various dynamic movements and running-based exercises of increasing intensity. This warm-up included two sets of following movements or exercises: Straight ahead, Hip Out, Hip in, circling partner, Shoulder contact, across the pitch, Bounding.

Lower leg stretches, upper body stretches, mobilization exercises, and stabilization exercises were performed before CMJ measurements were taken (directed by one of the researchers). Moreover, the participants performed three submaximal CMJ trials for familiarization before each CMJ measurement. CMJ testing was performed using previously used protocols^32^. Participants stood upright with the bar over their shoulders and applied pressure to prevent the bar from moving independently of their body. They were asked to jump as high as possible in a fluid movement.

According to previous studies, the test involved participants jumping as high as possible for each trial with a 400-g bar positioned on their shoulders along a horizontal plane. Similar to previous procedures^22,32,33^, subjects were encouraged to self-select the CMJ tension or rate with no attempt to standardize.

The participants were instructed not to pull their knees toward their bodies during the jumping phase. They were also instructed to stretch their legs and land with their legs straight during the flight phase. The participants performed three jumps, and jump performance was observed by the researcher. Recovery time between jumps was 2–3 min^32^. Any jump that was not performed following the proper technique was repeated. The best score (jump height) of the three jumps was recorded for evaluation.

As in previous research^20,22,33,34^, CMJ height and CMJ maximum velocity were used as the main criterion measures for neuromuscular fatigue. Device fixed to the floor during CMJ measurements and connected to the 400-g rod with a cable to be analyzed through an optical encoder (GymAware Power Tool, Kinetic Performance Technologies, Canberra, Australia). The players performed all CMJ tests between 17:00 and 17:15 in the afternoon. All testing sessions took place at the same time of day for each participant (± 15 min) and under similar environmental conditions (~ 23◦C ± 4 ◦C and ~ 60% ±  ~ 5% humidity).

### Match load monitoring

A friendly match was played for the players in official field conditions, with a 15-min rest between the two 45-min halves. During the friendly match, the players' heart rate, acceleration and distance were measured with the help of 10 Hz Polar Pro Team GPS (Polar Electro, Kempele, Finland). The internal and external load parameters obtained from the players during the match are as follows: *External load variables*; 00–13.99 km/h, 14–19.99 km/h and > 20 km/h (m), > 3 m s^2^ # Acc (N) values^35,36^, *Internal load variables*; % HRmax 50–59, % HRmax 60–69, % HRmax 70–79, % HRmax 80–89, % HRmax 90–100 (min)**.** It was ensured that the players played in the same tactical formation (4–4–2) in both halves of the match.

### Statistical analysis

Standard deviation and mean values for all parameters were reported. The normal distribution, Kolmogorov–Smirnov and homogeneity of all data obtained from the tests were tested by looking at the "skewness" and "kurtosis" values. The significance of temporal changes of fatigue measurements made at different hours was investigated by applying post hoc analysis of variance in repeated measurements. The effect sizes of the differences between the tests are shown by Cohen's d value. The effect size of the differences of the tests was determined as Cohen d, which was considered as 0–0.19 insignificant, 0.2–0.59 small, 0.6–1.19 medium, 1.20–1.99 large, 2.00–3.99 very large, and d > 4 excess^37^. Pearson analysis was chosen as the relationship since the data were normally distributed. As the level of the relationship: Minor (0.0), small (0.1), medium (0.3), large (0.5), very large (0.7), almost perfect (0.9) and perfect (1.0) levels were used. An alpha level of p < 0.05 was determined as the significance level^37^. All statistical analysis and graphs were made using R studio version 1.3.1093.

### Ethics approval and consent to participate

The study fully adheres to the ethical principles of the declaration of Helsinki as well as GCP guidelines. The study was approved by the Research Ethics Committee of Afyon Kocatepe University (Number: Dated 19.01.2021, numbered 26.01.2021-3764). Informed consent was obtained from all subjects agreed to participate in this study and answered the questionnaire.

## Results

Descriptive values for CMJ test are given in detail in Table 1. Table 2 shows the CMJ maximum velocity (m /s) and CMJ height velocity temporal variations. There was a statistically significant difference between the test 24 h before and the test after 24 h (p > 0.001, Cohen d; 1,210); 24 h before and the test after 24 h (p > 0.001, Cohen d; 1.578); 24 h before and the test after 48 h (p > 0.01, Cohen d; 0.922).

**Table 1:** Descriptive statistics.

Period tests	Mean	SD
After 120 h CMJ Maximum velocity (m/s)	3.21	0.343
After 24 h CMJ maximum velocity (m/s)	2.76	0.402
Before 24 h CMJ maximum velocity (m/s)	3.08	0.366
After 48 h CMJ maximum velocity (m/s)	3.06	0.353
After 72 h CMJ maximum velocity (m/s)	3.07	0.345
After 96 h CMJ maximum velocity (m/s)	3.17	0.436
After 120 h CMJ height (cm)	36.2	3.412
After 24 h CMJ height (cm)	33.0	3.410
Before 24 h CMJ height (cm)	36.1	4.154
After 48 h CMJ height (cm)	34.6	3.856
After 72 h CMJ height (cm)	36.1	4.626
After 96 h CMJ height (cm)	36.3	3.935

**Table 2:** Post Hoc comparisons—CMJ maximum velocity (m/sn) and CMJ height (cm).

Period tests		95% CI mean difference							
		Mean difference	Lower	Upper	Standard error	t	Cohen's d	p	
**CMJ maximum velocity (m/sn)**									
24 h before	24 h after	0.319	0.121	0.516	0.059	5.410	1.210	< 0.001	***
	48 h after	0.014	− 0.199	0.227	0.063	0.221	0.049	1.000	
	72 h after	0.009	− 0.108	0.127	0.035	0.272	0.061	1.000	
	96 h after	− 0.095	− 0.265	0.074	0.051	− 1.889	− 0.422	1.000	
	120 h after	− 0.129	− 0.363	0.106	0.070	− 1.837	− 0.411	1.000	
**CMJ height (cm)**									
24 h before	24 h after	3.050	1.601	4.499	0.432	7.059	1.578	< 0.001	***
	48 h after	1.450	0.271	2.629	0.352	4.125	0.922	0.009	**
	72 h after	− 0.050	− 1.415	1.315	0.407	− 0.123	− 0.027	1.000	
	96 h after	− 0.200	− 1.788	1.388	0.474	− 0.422	− 0.094	1.000	
	120 h after	− 0.100	− 1.749	1.549	0.492	− 0.203	− 0.045	1.000	

***p < 0.001, **p < 0.01.

**Figure 2:**
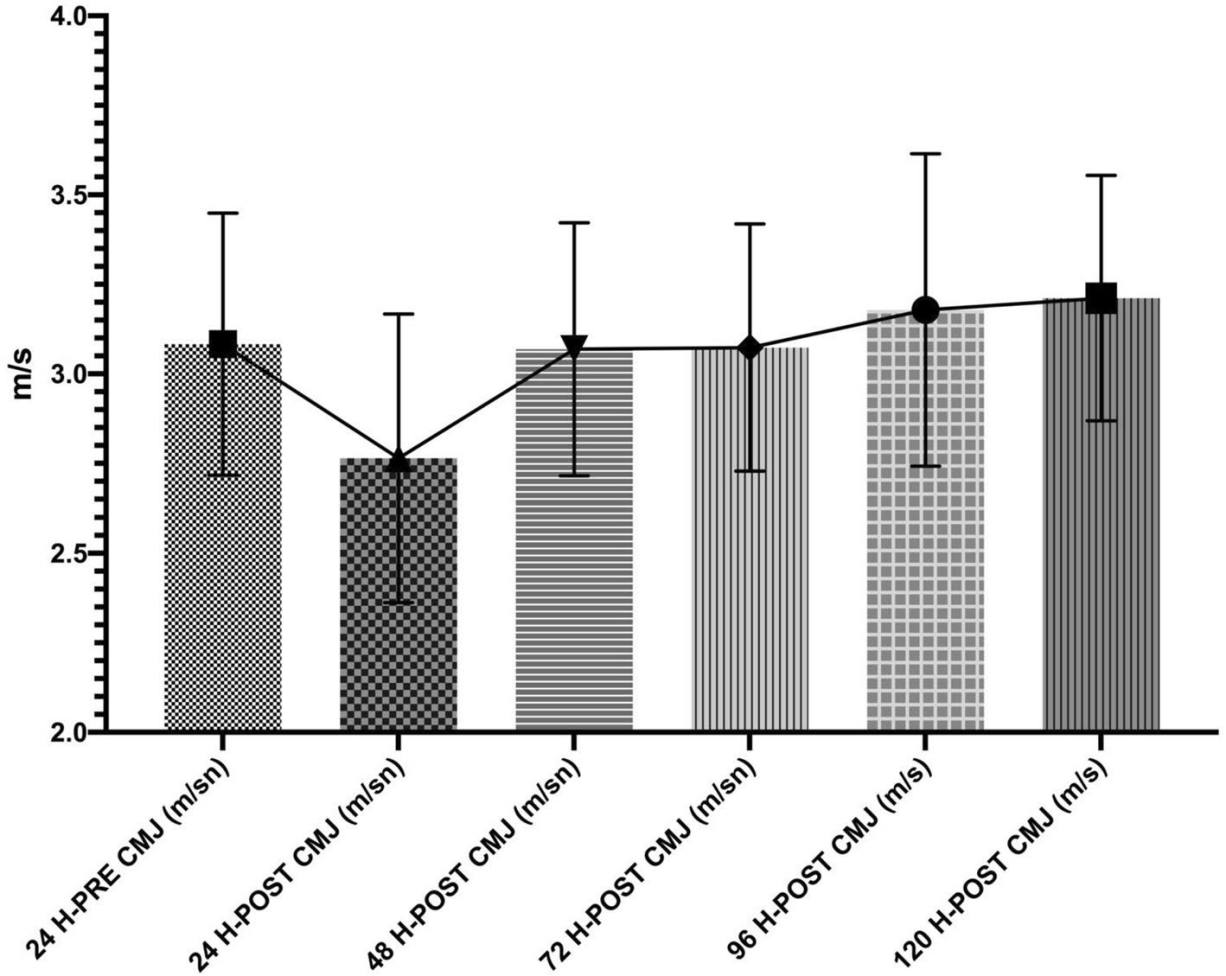
CMJ maximum velocity (m/s) values of the players at different measurement times.

**Figure 3:**
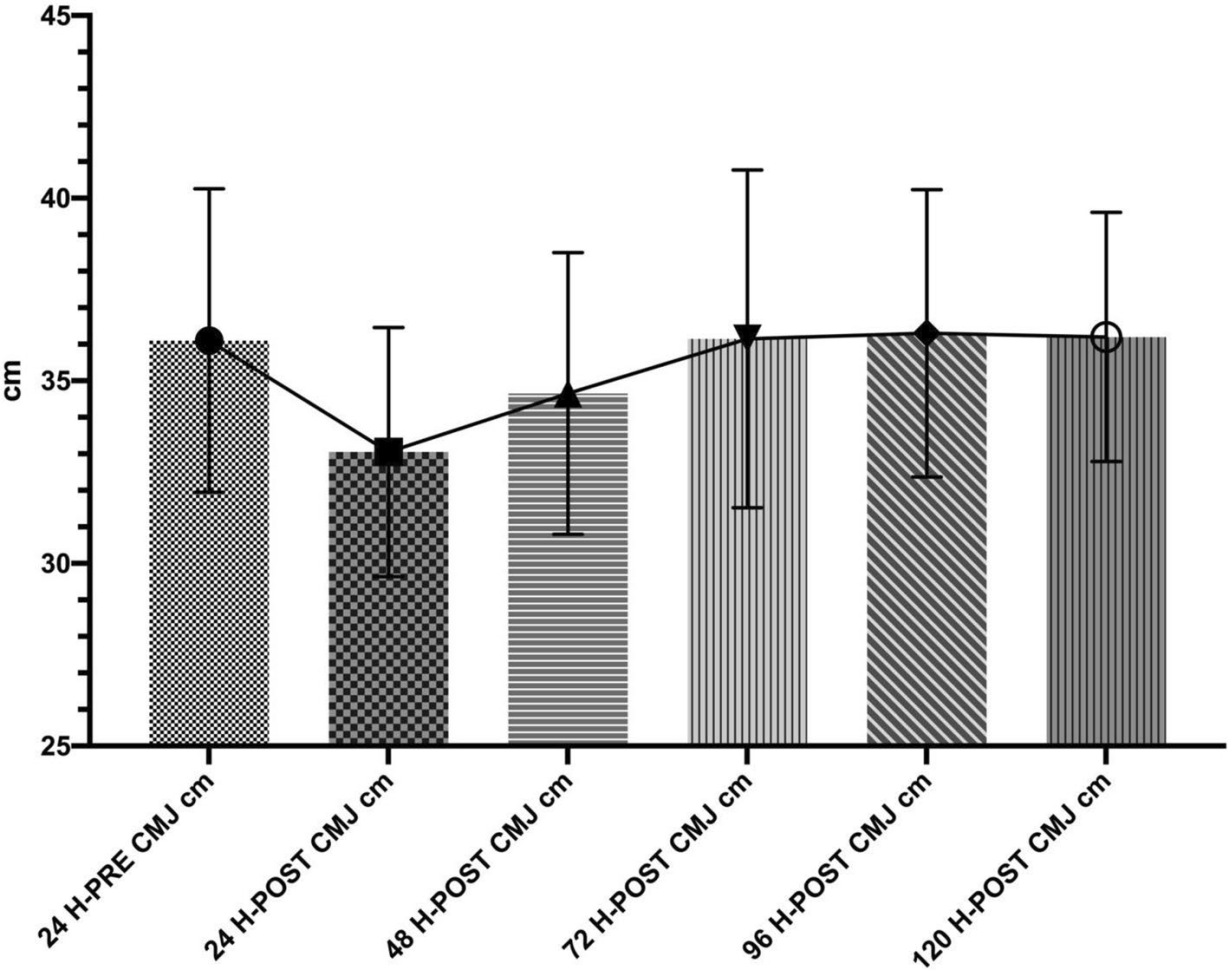
CMJ height (cm) values of the players at different measurement times.

Figure 2 shows the individual CMJ maximum velocity (m/s) values of the players at different measurement times. Figure 3 shows the individual CMJ height (cm) values of the players at different measurement times.

**Figure 4:**
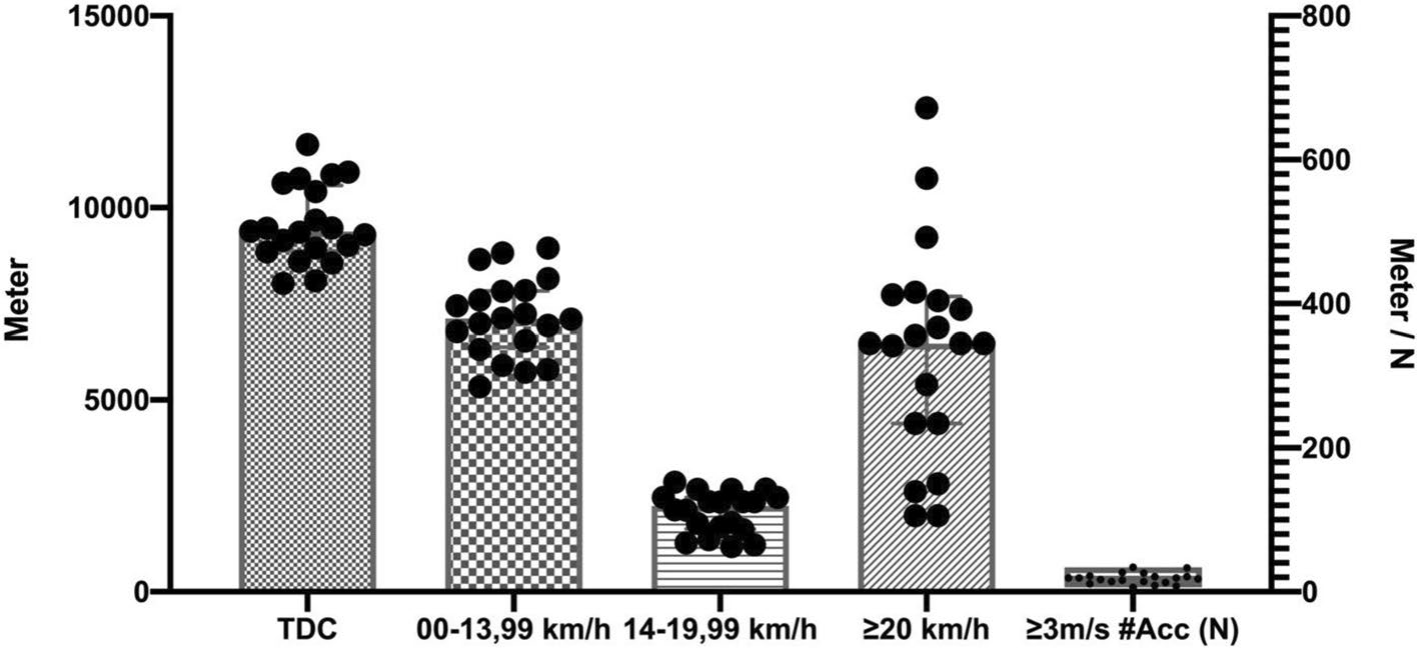
The total distance covered by all players in the match, over 00–13.99 km/h, 14–19.99 km/h and > 20 km/h (m) and ≥ 3 m/s #Acc (N).

**Figure 5:**
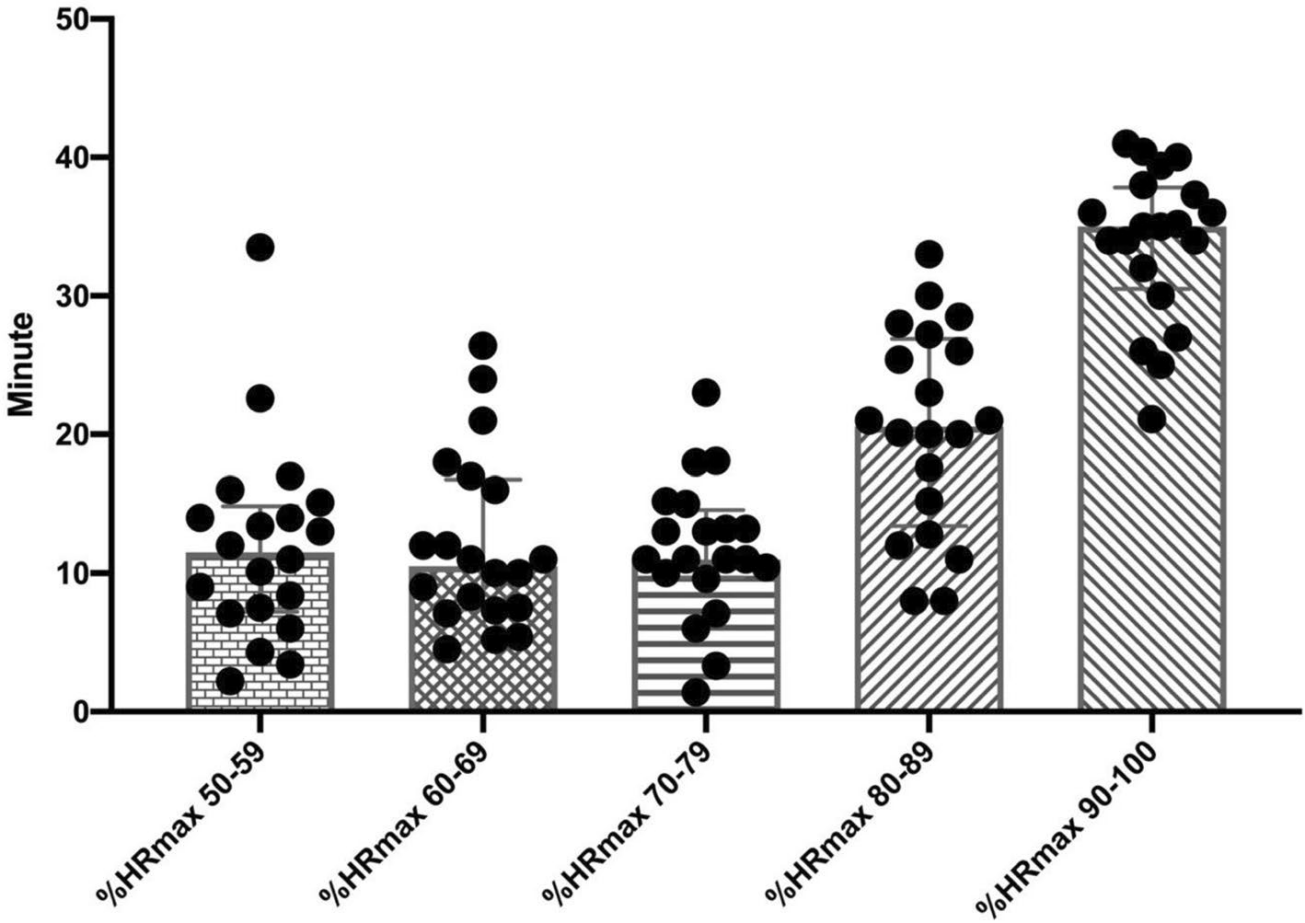
The time spent by all players during the match at% HRmax 50–59,% HRmax 60–69,% HRmax 70–79,% HRmax 80–89,% HRmax 90–100 (min).

In Fig. 4, the total distances covered by all players in the match, 0–13.99 km/h, 14–19.99 km/h and > 20 km/h and # Acc (N) values of all players > 3 m/s are shown (m). In Fig. 5, the time (min) spent by all players during the match in % Heartrate (HR) max 50–59, % HRmax 60–69, % HRmax 70–79, % HRmax 80–89, % HRmax 90–100.

Tables 3, 4, 5 and 6 show the relationship between the neuromuscular fatigue responses of the players at different times and the internal and external loads obtained from the match. As a result of the correlation analysis, no statistical relationship was found between any values.

**Table 3:** Comparison of the match external loads and CMJ heights variables.

Variable	TDC	00–13,99 km/h	V14–19,99 km/h	≥ 20 km/h	≥ 3 m/s #Acc (N)
**Before 24 h CMJ height (cm)**					
Pearson's r	− 0.224	− 0.318	0.345	− 0.536	− 0.298
p-value	0.342	0.172	0.136	0.015	0.202
Upper 95% CI	0.243	0.145	0.683	− 0.122	0.166
Lower 95% CI	− 0.606	− 0.667	− 0.115	− 0.791	− 0.654
**After 24 h CMJ height (cm)**					
Pearson's r	− 0.010	− 0.104	0.277	− 0.340	− 0.454
p-value	0.967	0.663	0.237	0.142	0.044
Upper 95% CI	0.435	0.355	0.641	0.121	− 0.014
Lower 95% CI	− 0.450	− 0.522	− 0.188	− 0.680	− 0.746
**After 48 h CMJ height (cm)**					
Pearson's r	− 0.154	− 0.242	0.309	− 0.461	− 0.181
p-value	0.518	0.304	0.185	0.041	0.446
Upper 95% CI	0.310	0.225	0.661	− 0.023	0.285
Lower 95% CI	− 0.558	− 0.618	− 0.155	− 0.750	− 0.577
**After 72 h CMJ height (cm)**					
Pearson's r	− 0.180	− 0.233	0.220	− 0.384	− 0.240
p-value	0.448	0.324	0.352	0.094	0.309
Upper 95% CI	0.285	0.234	0.604	0.070	0.227
Lower 95% CI	− 0.577	− 0.612	− 0.247	− 0.707	− 0.617
**After 96 h CMJ height (cm)**					
Pearson's r	− 0.063	− 0.103	0.225	− 0.518	− 0.083
p-value	0.791	0.665	0.340	0.019	0.728
Upper 95% CI	0.390	0.356	0.607	− 0.098	0.373
Lower 95% CI	− 0.492	− 0.522	− 0.241	− 0.782	− 0.507
**After 120 h CMJ height (cm)**					
Pearson's r	− 0.139	− 0.231	0.340	− 0.549	− 0.186
p-value	0.560	0.328	0.143	0.012	0.433
Upper 95% CI	0.324	0.236	0.680	− 0.141	0.280
Lower 95% CI	− 0.548	− 0.611	− 0.121	− 0.798	− 0.580

**Table 4:** Comparison of the match internal loads and CMJ heights variables.

Variable	%HRmax 50–59	%HRmax 60–69	%HRmax 70–79	%HRmax 80–89	%HRmax 90–100
**Before 24 h CMJ height (cm)**					
Pearson's r	0.176	0.191	− 0.071	− 0.151	− 0.180
p-value	0.457	0.421	0.766	0.526	0.448
Upper 95% CI	0.574	0.584	0.384	0.313	0.285
Lower 95% CI	− 0.289	− 0.275	− 0.498	− 0.556	− 0.577
**After 24 h CMJ height (cm)**					
Pearson's r	0.301	0.169	− 0.033	− 0.202	− 0.284
p-value	0.196	0.475	0.890	0.393	0.225
Upper 95% CI	0.656	0.569	0.416	0.264	0.181
Lower 95% CI	− 0.163	− 0.295	− 0.469	− 0.592	− 0.645
**After 48 h CMJ height (cm)**					
Pearson's r	0.078	0.004	− 0.013	0.030	− 0.134
p-value	0.745	0.986	0.957	0.901	0.572
Upper 95% CI	0.503	0.446	0.432	0.466	0.328
Lower 95% CI	− 0.378	− 0.439	− 0.453	− 0.418	− 0.544
**After 72 h CMJ height (cm)**					
Pearson's r	0.061	0.236	− 0.044	− 0.163	− 0.090
p-value	0.797	0.316	0.854	0.492	0.705
Upper 95% CI	0.491	0.615	0.406	0.301	0.367
Lower 95% CI	− 0.392	− 0.230	− 0.477	− 0.565	− 0.512
**After 96 h CMJ height (cm)**					
Pearson's r	0.049	0.019	− 0.035	− 0.002	− 0.050
p-value	0.837	0.936	0.883	0.992	0.833
Upper 95% CI	0.481	0.458	0.414	0.441	0.401
Lower 95% CI	− 0.402	− 0.427	− 0.470	− 0.444	− 0.482
**After 120 h CMJ height (cm)**					
Pearson's r	0.349	− 0.025	0.062	− 0.228	− 0.175
p-value	0.131	0.917	0.795	0.333	0.461
Upper 95% CI	0.686	0.422	0.491	0.238	0.290
Lower 95% CI	− 0.111	− 0.462	− 0.391	− 0.609	− 0.573

**Table 5:** Comparison of the match external loads and CMJ maximum velocity variables.

Variable	TDC	00–13,99 km/h	V14-19,99 km/h	≥ 20 km/h	≥ 3 m/s #Acc (N)
**Before 24 h CMJ maximum velocity (m/sn)**					
Pearson's r	0.216	0.199	0.035	− 0.054	0.314
p-value	0.360	0.401	0.883	0.820	0.178
Upper 95% CI	0.601	0.589	0.470	0.398	0.664
Lower 95% CI	− 0.250	− 0.267	− 0.414	− 0.485	− 0.149
**After 24 h CMJ maximum velocity (m/sn)**					
Pearson's r	0.240	0.182	0.085	0.046	0.367
p-value	0.308	0.443	0.722	0.849	0.112
Upper 95% CI	0.617	0.578	0.508	0.478	0.696
Lower 95% CI	− 0.227	− 0.283	− 0.372	− 0.405	− 0.091
**After 48 h CMJ maximum velocity (m/sn)**					
Pearson's r	0.121	− 0.052	0.279	0.177	− 0.012
p-value	0.611	0.829	0.234	0.456	0.959
Upper 95% CI	0.535	0.400	0.642	0.574	0.432
Lower 95% CI	− 0.340	− 0.483	− 0.187	− 0.288	− 0.452
**After 72 h CMJ maximum velocity (m/sn)**					
Pearson's r	0.329	0.314	0.014	− 0.022	0.353
p-value	0.156	0.177	0.953	0.925	0.127
Upper 95% CI	0.674	0.665	0.454	0.424	0.688
Lower 95% CI	− 0.133	− 0.149	− 0.431	− 0.460	− 0.106
**After 96 h CMJ maximum velocity (m/sn)**					
Pearson's r	0.301	0.251	0.063	0.052	0.203
p-value	0.197	0.285	0.791	0.829	0.391
Upper 95% CI	0.656	0.624	0.492	0.483	0.592
Lower 95% CI	− 0.163	− 0.215	− 0.390	− 0.400	− 0.263
**After 120 h CMJ maximum velocity (m/sn)**					
Pearson's r	0.219	0.135	0.084	0.232	0.230
p-value	0.354	0.570	0.724	0.325	0.328
Upper 95% CI	0.603	0.545	0.508	0.612	0.611
Lower 95% CI	− 0.248	− 0.327	− 0.372	− 0.235	− 0.236

**Table 6:** Comparison of the match internal loads and CMJ maximum velocity variables.

Variable	%HRmax 50–59	%HRmax 60–69	%HRmax 70–79	%HRmax 80–89	%HRmax 90–100
**Before 24 h maximum velocity (m/sn)**					
Pearson's r	− 0.009	− 0.201	0.111	0.168	− 0.085
p-value	0.968	0.395	0.642	0.480	0.723
Upper 95% CI	0.435	0.265	0.527	0.568	0.372
Lower 95% CI	− 0.450	− 0.591	− 0.349	− 0.297	− 0.508
**After 24 h CMJ maximum velocity (m/sn)**					
Pearson's r	0.115	− 0.267	− 0.114	0.232	− 0.052
p-value	0.630	0.254	0.633	0.325	0.829
Upper 95% CI	0.530	0.199	0.346	0.612	0.400
Lower 95% CI	− 0.345	− 0.635	− 0.530	− 0.235	− 0.483
**After 48 h CMJ maximum velocity (m/sn)**					
Pearson's r	0.203	− 0.051	0.027	0.039	− 0.284
p-value	0.390	0.832	0.909	0.871	0.224
Upper 95% CI	0.593	0.401	0.464	0.473	0.181
Lower 95% CI	− 0.263	− 0.482	− 0.420	− 0.411	− 0.646
**After 72 h CMJ maximum velocity (m/sn)**					
Pearson's r	− 0.046	− 0.375	0.069	0.304	0.017
p-value	0.847	0.103	0.773	0.192	0.944
Upper 95% CI	0.405	0.081	0.496	0.658	0.456
Lower 95% CI	− 0.479	− 0.701	− 0.385	− 0.160	− 0.429
**After 96 h CMJ maximum velocity (m/sn)**					
Pearson's r	0.126	− 0.242	0.225	0.121	− 0.256
p-value	0.595	0.303	0.340	0.612	0.277
Upper 95% CI	0.539	0.224	0.607	0.535	0.211
Lower 95% CI	− 0.335	− 0.619	− 0.241	− 0.340	− 0.627
**After 120 h CMJ maximum velocity (m/sn)**					
Pearson's r	− 0.082	− 0.263	0.132	0.362	− 0.201
p-value	0.732	0.263	0.580	0.117	0.395
Upper 95% CI	0.374	0.203	0.543	0.693	0.265
Lower 95% CI	− 0.506	− 0.632	− 0.330	− 0.096	− 0.591

## Discussion

In this study, the neuromuscular fatigue imposed on players after a football match was examined.

The results indicate a statistically significant difference between the test 24 h before and the test after 24 h (p > 0.001, Cohen d; 1,210); 24 h before and the test after 24 h (p > 0.001, Cohen d; 1.578); 24 h before and the test after 48 h (p > 0.01, Cohen d; 0.922).

The correlation analysis presents no statistical correlations between the internal and external load values obtained from the matches and the neuromuscular fatigue values measured at different time intervals.

Activities that occur within football matches cause fatigue in the players^38^. The fatigue variables triggered by matches directly affect the structure of the training program during the next days, as well as the match to be held the following week^15^. The fatigue of the athletes is controlled during the trainings and it is ensured that they play the matches in a recovered way^15,38,39^. The effective control of fatigue is provided by sports scientists, coaches, or strength conditioning coaches^40^. Keeping fatigue under control and promoting optimum performance from players are related to the ability to balance fatigue, nutrition and rest^15,40^. The balance of the fatigue-resting mechanism in football teams is carried out by individuals responsible for monitoring the training load and fatigue of the team^40,41^.

The fatigue that occurs in the players during training and matches is evaluated under two different load types^15,40,42,43^. Physiological stressors that occur in players during training and matches are called internal load^15,40,42,43^. Kinematic activities that create physiological stressors are also called external loads. In our study, heart rate variables are called internal load, and distances covered are called external load^15,40,42,43^.

It is of vital importance for the players that these individuals determine how much fatigue occurs during training and matches and take appropriate action^43^. The main problem addressed in our study is related to temporal changes in neuromuscular fatigue during the week, which were determined by measuring neuromuscular fatigue that occurs after the match through CMJ tests performed at different times.

Decreased neuromuscular performance capacity is generally reported to be associated with muscle fatigue^40,42^. In previous studies, significant decreases in CMJ performance were observed after football matches^5,44^. Studies measuring the height of the CMJ test have reported that jump height decreases with fatigue^24,28,44–46^. Prolonged and repetitive exercises affect the SSC and may reduce CMJ performance in this case^28,33,38,44^. Studies that have investigated the performance of SSC on neuromuscular fatigue in more detail show changes in strength development rates in different milliseconds and their relationships with maximum power, average power, and biochemical parameters, as well as the sensitivity of SSC in showing such neuromuscular fatigue^45,47,48^. The jump heights obtained via the CMJ test are relatively precise and, thus, are given great importance in the literature^45,49^. Researchers have also stated that these are valid and reliable tests to measure the fatigue associated with various activities, including common football activities, in which the function of the lower extremity is predominant^24,28,31,42,44,45,47,49,50^.

In our study, it was found that the match triggered neuromuscular fatigue. This fatigue generally decreased 24 h and 48 h after the match. The heights achieved in the CMJ test at the 24th and 48th hours after the match was significantly lower than the pre-match jump heights. A statistically significant difference was recorded between jump heights 24 h before the match and 24 h after the match (p > 0.001, Cohen’s d = 1.578). A statistically significant difference was also found in jump heights 24 h before the match as well as 48 h after (p > 0.01, Cohen’s d = 0.922). Previous studies^5,6,51,52^ support our findings—In these studies, CMJ heights decreased at the 24th and 48th hours after a match before increasing.

CMJ test performance might not be affected by the physiological neuromuscular fatigue that arises as a result of the affection of SSC by fatigue^20^. The maximum velocity values examined in our study during CMJ might complicate the measurement of neuromuscular fatigue^18^. Studies that have aimed to determine neuromuscular fatigue imposed on players after matches have considered the influence of maximum velocity during the CMJ test, similar to our study^20,22,33^. The maximum velocity parameter in the CMJ test is significantly different 24 h after a match compared to the pre-match level. There is also a statistically significant difference between the test 24 h before and 24 h after the match (p > 0.001, Cohen’s d = 1,210), researchers found that neuromuscular fatigue occurred after a match and decreased the maximum velocity attained during a CMJ test, which supports our results^20,22,33^.

The values obtained from the tests we used to measure fatigue may be random. As a concrete example, the maximum velocity values in our CMJ test may have determined the fatigue in one measurement randomly while remaining undetected for the other measurement. Therefore, the reliability of the measured values must be determined in order to understand whether the measured values give random data. The reliability of both CMJ tests used in our study to measure fatigue was reported to be high in previous studies^22,31,33,47,49,50^. The relationship between the internal and external game loads on the players during the game and the neuromuscular fatigue measurements seems statistically insignificant. In order to clarify this situation, it is thought that the relationship of more matches with neuromuscular fatigue measurements should be investigated in the future. Similar to our findings, some studies^29,52^ did not find a relationship between match internal and external loads and neuromuscular fatigue values.

There are some negative relations between internal and external loads and CMJ maximum velocity (m/s) values in the match, but these relations are statistically insignificant. Likewise, there are negative relations between internal and external loads and CMJ heights (cm) during the competition, but these relations are statistically insignificant. In general, as the activities performed during the match increase, the CMJ maximum velocity (m/s) and CMJ heights (cm) values decrease.

It is generally thought that fatigue affects the acceleration ability of the muscle during jumping, which, in turn, causes a decrease in velocity. The limitations of this study were that it examined fatigue changes over a one-week period and was conducted with a limited number of players. In future studies, it is recommended that more players are involved so that differences between playing positions can be examined alongside the effect of weekly match changes on fatigue during the following week and changes in different periods of the season.

## Conclusion

The neuromuscular fatigue imposed on football players after a match caused statistically significant differences in CMJ test performance before and after the match. Specifically, the jump heights obtained from the CMJ test before the match differed from the values recorded 24 and 48 h after the match. Likewise, it was found that the maximum velocity values in the CMJ test recorded 24 h after the match were different from the values recorded before the match, the neuromuscular fatigue values at the 24th hour after the match. Unlike with jump height, no significant difference was found between the pre-match values and the values recorded 48 h after the match. Meanwhile, no statistical correlation was found between the neuromuscular fatigue values of the players obtained at different time periods and the internal and external load values recorded during the match. It should be noted that fatigue continues until the after the match. Considering the effects of fatigue on players, complex situations such as adequate rest, nutrition, balanced training loads, effective training periodization, the appropriate selection of players to compete in the current week’s match, and the determination of rest days during the week can create a road map for sports scientists, trainers, and strength conditioning coaches in light of the findings of this study.

## Data Availability

The datasets generated during and analyzed during the current study are available from Z.A on reasonable request.

## References

[1] Hader K (2019). Monitoring the athlete match response: Can external load variables predict post-match acute and residual fatigue in soccer? A systematic review with meta-analysis. Sport. Med. Open.

[2] Reilly T, Drust B, Clarke N (2008). Muscle fatigue during football match-play. Sports Med..

[3] Julian R, Page RM, Harper LD (2021). The effect of fixture congestion on performance during professional male soccer match-play: A systematic critical review with meta-analysis. Sport. Med..

[4] Garcia GR (2022). Effects of congested fixture and matches’ participation on internal and external workload indices in professional soccer players. Sci. Rep..

[5] Silva JR (2018). Acute and residual soccer match-related fatigue: A systematic review and meta-analysis. Sport. Med..

[6] Silva JR (2013). Neuromuscular function, hormonal and redox status and muscle damage of professional soccer players after a high-level competitive match. Eur. J. Appl. Physiol..

[7] Owen AL, Djaoui L, Newton M, Malone S, Mendes B (2017). A contemporary multi-modal mechanical approach to training monitoring in elite professional soccer. Sci. Med. Footb..

[8] Gabbett TJ (2016). The training—injury prevention paradox: should athletes be training smarter and harder?. Br. J. Sports Med..

[9] Gandevia SC (2001). Spinal and supraspinal factors in human muscle fatigue. Physiol. Rev..

[10] Bigland-Ritchie B, Woods JJ (1984). Changes in muscle contractile properties and neural control during human muscular fatigue. Muscle Nerve.

[11] Meeusen R (2004). Hormonal responses in athletes: the use of a two bout exercise protocol to detect subtle differences in (over)training status. Eur. J. Appl. Physiol..

[12] Komi PV (2000). Stretch-shortening cycle: A powerful model to study normal and fatigued muscle. J. Biomech..

[13] Nicol C, Avela J, Komi PV (2006). The stretch-shortening cycle. Sport. Med..

[14] Horita T, Komi PV, Nicol C, Kyröläinen H (1999). Effect of exhausting stretch-shortening cycle exercise on the time course of mechanical behaviour in the drop jump: Possible role of muscle damage. Eur. J. Appl. Physiol..

[15] Carling C (2018). Monitoring of post-match fatigue in professional soccer: Welcome to the real world. Sport. Med..

[16] Andersson H (2008). Neuromuscular fatigue and recovery in elite female soccer: Effects of active recovery. Med. Sci. Sports Exerc..

[17] Russell M (2016). Relationships between match activities and peak power output and Creatine Kinase responses to professional reserve team soccer match-play. Hum. Mov. Sci..

[18] Claudino JG (2017). The countermovement jump to monitor neuromuscular status: A meta-analysis. J. Sci. Med. Sport.

[19] McLellan CP, Lovell DI, Gass GC (2011). Markers of postmatch fatigue in professional rugby league players. J. Strength Cond. Res..

[20] Marrier B (2017). Quantifying neuromuscular fatigue induced by an intense training session in rugby sevens. Int. J. Sports Physiol. Perform..

[21] Robineau J, Jouaux T, Lacroix M, Babault N (2012). Neuromuscular Fatigue Induced by a 90-Minute Soccer Game Modeling. J. Strength Cond. Res..

[22] Garrett JM (2020). Comparison of a countermovement jump test and submaximal run test to quantify the sensitivity for detecting practically important changes within high-performance Australian rules football. Int. J. Sports Physiol. Perform..

[23] Wu PP-YY (2019). Predicting fatigue using countermovement jump force-time signatures: PCA can distinguish neuromuscular versus metabolic fatigue. PLoS ONE.

[24] Gathercole R, Sporer B, Stellingwerff T, Sleivert G (2015). Alternative countermovement-jump analysis to quantify acute neuromuscular fatigue. Int. J. Sports Physiol. Perform..

[25] de Hoyo M (2016). Influence of football match time–motion parameters on recovery time course of muscle damage and jump ability. J. Sports Sci..

[26] Thorpe RT (2017). The influence of changes in acute training load on daily sensitivity of morning-measured fatigue variables in elite soccer players. Int. J. Sports Physiol. Perform..

[27] Thorpe RT (2015). Monitoring fatigue during the in-season competitive phase in elite soccer players. Int. J. Sports Physiol. Perform..

[28] Nedelec M (2014). The influence of soccer playing actions on the recovery kinetics after a soccer match. J. Strength Cond. Res..

[29] Varley I, Lewin R, Needham R, Thorpe RT, Burbeary R (2017). Association between match activity variables, measures of fatigue and neuromuscular performance capacity following elite competitive soccer matches. J. Hum. Kinet..

[30] Shearer DA (2017). Measuring recovery: An adapted Brief Assessment of Mood (BAM+) compared to biochemical and power output alterations. J. Sci. Med. Sport.

[31] Markovic G, Dizdar D, Jukic I, Cardinale M (2004). Reliability and factorial validity of squat and countermovement jump tests. J. Strength Cond. Res..

[32] Taylor K-L, Cronin J, Gill ND, Chapman DW, Sheppard J (2010). Sources of variability in iso-inertial jump assessments. Int. J. Sports Physiol. Perform..

[34] Garrett J (2019). A novel method of assessment for monitoring neuromuscular fatigue in Australian rules football players. Int. J. Sports Physiol. Perform..

[34] Buchheit M, Lacome M, Cholley Y, Simpson BM (2018). Neuromuscular responses to conditioned soccer sessions assessed via GPS-embedded accelerometers: Insights into tactical periodization. Int. J. Sports Physiol. Perform..

[37] Sweeting AJ, Cormack SJ, Morgan S, Aughey RJ (2017). When is a sprint a sprint? A review of the analysis of team-sport athlete activity profile. Front. Physiol..

[38] Malone JJ, Lovell R, Varley MC, Coutts AJ (2017). Unpacking the black box: Applications and considerations for using GPS Devices in Sport. Int. J. Sports Physiol. Perform..

[39] Hopkins WG (2009). Progressive statistics for studies in sports medicine and exercise science. Med. Sci. Sports Exerc..

[38] Mohr M, Krustrup P, Bangsbo J (2005). Fatigue in soccer: A brief review. J. Sports Sci..

[39] Silva AF, Conte D, Clemente FM (2020). Decision-making in youth team-sports players: A systematic review. Int. J. Environ. Res. Public Health.

[40] Halson SL (2014). Monitoring training load to understand fatigue in athletes. Sport. Med..

[41] Weston M (2018). Training load monitoring in elite English soccer: a comparison of practices and perceptions between coaches and practitioners. Sci. Med. Footb..

[44] Thorpe RT, Atkinson G, Drust B, Gregson W (2017). Monitoring fatigue status in elite team-sport athletes: Implications for practice. Int. J. Sports Physiol. Perform..

[43] Djaoui L, Haddad M, Chamari K, Dellal A (2017). Monitoring training load and fatigue in soccer players with physiological markers. Physiol. Behav..

[44] Brownstein CG (2017). Etiology and recovery of neuromuscular fatigue following competitive soccer match-play. Front. Physiol..

[45] Gathercole RJ, Sporer BC, Stellingwerff T, Sleivert GG (2015). Comparison of the capacity of different jump and sprint field tests to detect neuromuscular fatigue. J. Strength Cond. Res..

[48] McHugh MP (2019). Countermovement jump recovery in professional soccer players using an inertial sensor. Int. J. Sports Physiol. Perform..

[47] Lombard W, Reid S, Pearson K, Lambert M (2017). Reliability of metrics associated with a counter-movement jump performed on a force plate. Meas. Phys. Educ. Exerc. Sci..

[48] Mclellan CP, Lovell DI, Gass GC (2011). The role of rate of force development on vertical jump performance. J. Strength Cond. Res..

[51] Roe G (2016). Between-days reliability and sensitivity of common fatigue measures in rugby players. Int. J. Sports Physiol. Perform..

[50] Heishman AD (2020). Countermovement jump reliability performed with and without an arm swing in NCAA division 1 intercollegiate basketball players. J. Strength Cond. Res..

[53] Silva R, Clemente FM, González-Fernández FT, Bernardo A, Ardigò LP (2021). Weekly variations of short-duration maximal jumping performance in soccer players: Exploring relationships with accumulated training load and match demands. Front. Physiol..

[52] Rowell AE, Aughey RJ, Hopkins WG, Stewart AM, Cormack SJ (2017). Identification of sensitive measures of recovery after external load from football match play. Int. J. Sports Physiol. Perform..

